# Double-Negative T-Cells during Acute Human Immunodeficiency Virus and Simian Immunodeficiency Virus Infections and Following Early Antiretroviral Therapy Initiation

**DOI:** 10.3390/v16101609

**Published:** 2024-10-14

**Authors:** Alexis Yero, Tao Shi, Julien A. Clain, Ouafa Zghidi-Abouzid, Gina Racine, Cecilia T. Costiniuk, Jean-Pierre Routy, Jérôme Estaquier, Mohammad-Ali Jenabian

**Affiliations:** 1 Department of Biological Sciences and CERMO-FC Research Centre, Université du Québec à Montréal (UQAM), Montreal, QC H2X 3X8, Canada; yero_diaz.alexis@courrier.uqam.ca (A.Y.); shhm2001@gmail.com (T.S.); 2 Centre Hospitalier Universitaire (CHU) de Québec Centre de Recherche, Faculté de Médecine, Université Laval, Québec, QC G1V 0A6, Canada; jclain2@gmail.com (J.A.C.); ouafa.zghidi-abouzid@crchudequebec.ulaval.ca (O.Z.-A.); gina.racine@crchudequebec.ulaval.ca (G.R.); jerome.estaquier@crchudequebec.ulaval.ca (J.E.); 3 Infectious Diseases and Immunity in Global Health Program, Research Institute of McGill University Health Centre, Montreal, QC H3H 2R9, Canada; cecilia.costiniuk@mcgill.ca (C.T.C.); jean-pierre.routy@mcgill.ca (J.-P.R.); 4 Chronic Viral Illness Service, Division of Infectious Disease, Department of Medicine, McGill University Health Centre, Montreal, QC H4A 3J1, Canada; 5 Department of Microbiology, Infectiology and Immunology, Faculty of Medicine, Université de Montréal, Montreal, QC H3T 1J4, Canada

**Keywords:** CD4^−^CD8^−^ T-cells, double negative (DN) T-cells, acute SIV infection, acute HIV infection, early ART, regulatory T-cells (Tregs)

## Abstract

HIV infection significantly affects the frequencies and functions of immunoregulatory CD3^+^CD4^−^CD8^−^ double-negative (DN) T-cells, while the effect of early antiretroviral therapy (ART) initiation on these cells remains understudied. DN T-cell subsets were analyzed prospectively in 10 HIV+ individuals during acute infection and following early ART initiation compared to 20 HIV-uninfected controls. In this study, 21 Rhesus macaques (RMs) were SIV-infected, of which 13 were assessed during acute infection and 8 following ART initiation four days post-infection. DN T-cells and FoxP3^+^ DN Treg frequencies increased during acute HIV infection, which was not restored by ART. The expression of activation (HLA-DR/CD38), immune checkpoints (PD-1/CTLA-4), and senescence (CD28^−^CD57^+^) markers by DN T-cells and DN Tregs increased during acute infection and was not normalized by ART. In SIV-infected RMs, DN T-cells remained unchanged despite infection or ART, whereas DN Treg frequencies increased during acute SIV infection and were not restored by ART. Finally, frequencies of CD39^+^ DN Tregs increased during acute HIV and SIV infections and remained elevated despite ART. Altogether, acute HIV/SIV infections significantly changed DN T-cell and DN Treg frequencies and altered their immune phenotype, while these changes were not fully normalized by early ART, suggesting persistent HIV/SIV-induced immune dysregulation despite early ART initiation.

## 1. Introduction

Double negative (DN) CD3^+^CD4^−^CD8^−^ T-cells, are a unique population of T lymphocytes lacking CD4 and CD8 co-receptor expression, comprising around 1–5% of T-cells circulating in the peripheral blood [1,2,3,4]. DN T-cells can originate from both thymic and extrathymic sources. Thymocytes may escape negative selection in the thymus during T-cell development without upregulating CD4 or CD8 receptor molecules [5,6]. DN T-cells also derive from mature CD4 and CD8 T-cells that downregulate their expression levels in the periphery [7,8,9,10].

DN T-cell subsets, known as T helper (Th)-like DN T-cells, produce effector cytokines, including IL-4, IL-17, and IFN-γ, which are commonly expressed by their counterpart CD4 Th cells Th2, Th17, and Th1 [7,11]. Consequently, they can drive inflammatory processes and contribute to the pathogenesis of certain autoimmune disorders, including systemic lupus erythematosus (SLE) and Sjögren’s syndrome [12,13]. However, other groups have reported that DN T-cells exhibit regulatory and immunosuppressive functions similar to regulatory T-cells (Tregs), known as DN regulatory T-cells (DN Tregs), by producing immunosuppressive cytokines such as IL-10 and transforming growth factor-beta 1 (TGF-β1) [14,15,16]. DN T-cells can also produce cytotoxic molecules such as perforin and granzyme B [17,18,19] and induce apoptosis in target cells via the Fas/FasL pathway [20]. Therefore, DN T-cells represent a complex population of CD3 T-cells with both pro- and anti-inflammatory properties. 

Human immunodeficiency virus (HIV) proteins such as *Nef*, *Vpu*, and *Env* can also downregulate CD4 expression on infected CD4 T-cells, generating peripheral DN T-cells [8,21,22,23,24,25,26]. In simian immunodeficiency virus (SIV) infection models in African green monkeys (AGMs), it was also demonstrated that the epigenetic regulation of CD4 receptors promotes CD4 downregulation [27,28], and that homeostatic cytokines such as IL-2 can also contribute to decreased CD4 expression [29,30]. Moreover, the conversion of CD4^+^ T-cells into virus-resistant CD4^−^ T-cells that maintain CD4-like functions has also been reported in SIV-infected AGMs [30,31,32,33]. Importantly, persistent antibody-mediated CD4 T-cell depletion was not associated with gut dysfunction in SIV-infected AGMs, indicating that disease progression is independent of CD4 T-cell restoration in these animals [34]. In addition, CD8 downregulation in CD8 T-cells can also contribute to the pool of DN T-cells [35]. We and others have demonstrated that DN T-cells could serve as viral reservoirs and harbor replication-competent HIV provirus, contributing to HIV persistence in antiretroviral therapy (ART)-treated people with HIV (PWH) [19,36,37,38]. It has been reported that the relative frequency of DN T-cells is increased in the blood of PWH, particularly those with advanced disease and low CD4 T-cell counts [39]. However, other groups found that the absolute counts of DN T-cells are lower in PWH compared to uninfected individuals [40], and this frequency is reduced in PWH with high viral loads (VLs) during early infection [41] as well in immunological non-responders despite prolonged ART [42]. In a pathogenic model of SIV infection (rhesus macaque, RM), the frequencies of DN T-cells have been reported to be similar to uninfected RMs but express higher levels of apoptotic markers [43], whereas in pigtailed macaques, a modest increase in DN T-cells was observed after SIV infection [44]. In contrast, in a nonpathogenic SIV-infected model (sooty mangabey), DN T-cells displayed helper T-cell functions and proliferative capacity [33,45,46]. These differences in DN T-cell dynamics could also reflect the emergence of DN Tregs. Indeed, DN T-cells expressing the transcription factor FoxP3 are associated with disease progression along with increased expression of CD39 and CD25 and increased proliferative capacity (Ki67 expression) [47]. Thus, the frequency of DN Tregs correlates negatively with CD4 T-cell counts and the CD4/CD8 ratio and positively with immune activation and systemic inflammation [47]. 

However, the impact of early ART imitation on the dynamics of DN T-cells and their regulatory subset is understudied. Thus, here, we assessed the changes in DN T-cells and DN Tregs during both acute HIV and SIV infections and evaluated the effect of early ART initiation on these cells.

## 2. Materials and Methods

### 2.1. Ethical Considerations for Human Study

This study, which followed the principles of the Helsinki Declaration, was approved by the Ethical Review Board of the Université du Québec à Montréal (UQAM) under protocol number #2014-452(approved on 10 August 2015). Before blood collection, all participants provided written informed consent for research-oriented biobanking purposes.

### 2.2. Human Study Population

A total of 30 individuals were enrolled in our study, including 10 PWH in acute infection [median: 100 days post-infection and interquartile range (IQR): 46–140 days] and 20 HIV non-infected controls (Table 1). PWH were longitudinally assessed during viremia in acute infection and following ART initiation during early infection [median: 165 days post-infection and IQR: 97–212 days; median duration of ART: 1.72 years and IQR: 1.43–2 years]. In the ART-treated group, 9 out of 10 individuals reached undetectable VL following early ART initiation (Table S1). The median nadir CD4 T-cell count for PWH was 258.5 cells/μL and IQR: 207.5–530 cells/μL. Peripheral blood mononuclear cells (PBMCs) from PWH and uninfected controls were obtained from the Montreal Primary HIV Infection cohort. Blood sample collection coincided with the estimated infection duration in both acute and ART-treated groups. Additionally, for the ART-treated group, sample collection aligned with the treatment duration. Peripheral blood was collected in EDTA-containing collection tubes, followed by PBMC isolation by Ficoll (Wisent, Montreal, QC, Canada) centrifugation. Isolated PBMCs were cryopreserved in liquid nitrogen to be analyzed in batches. All participants in this study tested negative for other prevalent sexually transmitted infections (STIs) as well as HCV and HBV.

### 2.3. Ethical Considerations for RM Study 

All monkeys were accommodated at Laval University’s non-human primate (NHP) facilities in Quebec City, Quebec, Canada, adhering to the guidelines of the Canadian Council on Animal Care (http://www.ccac.ca, accessed on 29 September 2024). This study received approval from the Laval University Animal Protection Committee (#106004). The monkeys’ diet consisted of standard monkey chow supplemented with daily portions of fruit, vegetables, and unlimited access to water. Veterinary staff administered and supervised social enrichment activities while the animals’ overall health was monitored daily. Any animals exhibiting significant distress, illness, or weight loss underwent clinical evaluation and, if necessary, were humanely euthanized using barbiturates, following the Veterinary Medical Association’s guidelines.

### 2.4. Experimental SIV Infection Protocol 

**Figure 1:**
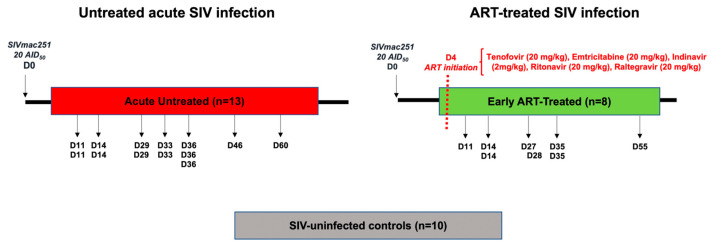
Study protocol. A total of 21 female Rhesus macaques (RMs) were infected intravenously with 20 50% animal infectious doses (AIDs) of SIVmac251 virus, and the specimens were collected in the acute phase of infection in 13 animals in the absence of ART. Eight monkeys were treated four days after the infection in a daily manner with an ART cocktail. Blood specimens were obtained from 10 SIV-uninfected animals that were used as controls. Black arrows represent the time when samples from whole blood were taken. Of note, each “D” followed by a number indicates one animal; therefore, in some cases, blood was collected from more than one animal on the same day. *Nota bene*: Blood samples from 3 animals were collected before and after SIV infection.

Thirty-one female RMs, all negative for SIVmac, STLV-1 (Simian T Leukemia Virus type-1), SRV-1 (type D retrovirus), and herpes-B viruses, were included in our study (Table 2). Of these, 21 animals were infected intravenously with 20 AID_50_ of SIVmac251 virus, while only 8 of them received early ART treatment initiated four days post-infection consisting of a combination of reverse transcriptase inhibitors Tenofovir (20 mg/kg) and Emtricitabine (20 mg/kg), protease inhibitors Indinavir (2 mg/kg) and Ritonavir (20 mg/kg), as well as integrase inhibitor Raltegravir (20 mg/kg) [48,49] (Figure 1). Seven early ART-treated animals reached undetectable VL following treatment, and in only one animal, we detected SIV RNA (Table S2). The remaining 13 animals remained untreated during the acute phase of the infection (11–60 days post-infection). Blood specimens from 10 SIV-uninfected RMs were also included as a control (Figure 1). Blood samples were collected in EDTA collection tubes and frozen at −80 °C until further use. Samples were later defrosted and used for flow cytometry analysis.

### 2.5. Flow Cytometry Analysis

Multi-color flow cytometry was performed on frozen whole blood in the RM study and frozen PBMCs in the human study. Dead cells were excluded using the LIVE/DEAD Fixable Aqua Dead Cell Stain Kit (Invitrogen, MA, USA). Surface staining was conducted for 1 h at 4 °C in PBS + 2% fetal bovine serum (FBS). Cells were fixed and permeabilized for 40 min at 4 °C upon surface staining using the Transcription Factor Buffer Set (BD Bioscience, Franklin Lakes, NJ, USA). Intracellular staining for FoxP3 and CTLA-4 was performed for 1 h at 4 °C in a Perm/Wash solution (BD Bioscience, Franklin Lakes, NJ, USA). Data were acquired using a 3-laser BD Fortessa X-20 cytometer and analyzed using FlowJo v10.9.0 (Ashland, OR, USA). The antibodies used for immune phenotyping are listed in Table 3.

### 2.6. Statistical Analysis

Statistical analysis was performed using GraphPad Prism V10 (Boston, MA, USA). The Kolmogorov–Smirnov test was employed to assess variable distribution. The Kruskal–Wallis test was used to identify significant differences among more than two research groups. The Mann–Whitney rank test for unpaired variables determined differences between various animal groups. The Wilcoxon matched-pairs signed rank test was used to compare paired variables. The Spearman rank correlation test was also applied to detect associations among study variables. The figures include only statistical significances (*, *p* < 0.05; **, *p* < 0.01; ***, *p* < 0.001; ****, *p* < 0.0001).

## 3. Results

### 3.1. Frequencies of DN T-Cells Increasing during Early HIV Infection and Not Being Restored by Early ART 

**Figure 2:**
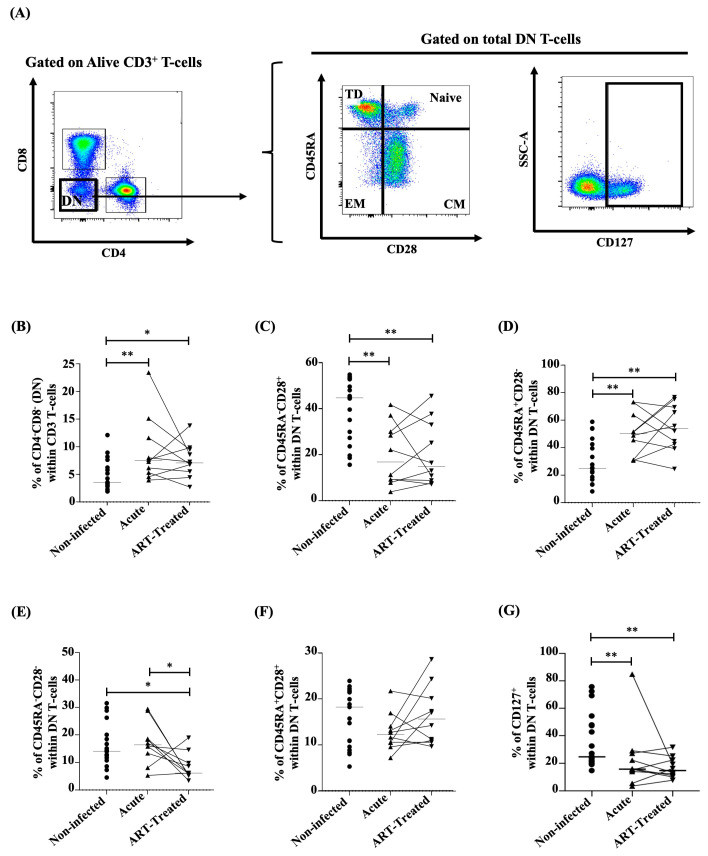
(**A**) Gating strategy used in flow cytometry to determine total CD3^+^CD4^−^CD8^−^ (double-negative, DN) T-cell (left) and DN T-cell memory subsets based on CD45RA/CD28 and CD127 expression (right) in the human study. Percentages determined in flow cytometry of total DN T-cells (**B**), central memory (CM, CD45RA^−^CD28^+^) (**C**), terminally differentiated (TD, CD45RA^+^CD28^−^) (**D**), effector memory (EM, CD45RA^−^CD28^−^) (**E**), and naïve (CD45RA^+^CD28^+^) (**F**) subsets within DN T-cells in the human study. (**G**) Percentages determined in flow cytometry of CD127^+^ DN T-cells in the human study. After the Kruskal–Wallis analysis, the differences among the three study groups were determined by a nonparametric Mann–Whitney rank test for unpaired variables (non-infected vs. acute, non-infected vs. ART-treated) and a Wilcoxon signed-rank test for paired variables (acute vs. ART-treated). Sample sizes in flow cytometry analysis: non-infected (*n* = 20), acute, and ART-treated (*n* = 10). Horizontal lines in graphs represent the median. Only statistical significances are presented (*, *p* < 0.05; **, *p* < 0.01).

The relative frequencies of total DN T-cells (CD3^+^CD4^−^CD8^−^) were significantly higher during early HIV infection but not normalized after early ART initiation (Figure 2A,B) compared to non-infected controls and positively correlated with CD8 T-cell counts (Table 4). We also found a switch in the DN T-cell differentiation phenotype that was associated with lower frequencies of central memory (CM, CD45RA^−^CD28^+^) and higher frequencies of terminally differentiated (TD, CD45RA^+^CD28^−^) subsets vs. non-infected controls (Figure 2A,C,D). This profile was not restored by ART initiation in the early phase of infection, but the effector memory (EM, CD45RA^−^CD28^−^) subset was decreased in early ART-treated individuals (Figure 2A,C–E). The frequency of CM DN T-cells was negatively associated with CD8 T-cell counts and the duration of treatment, while a positive association was found with the CD4/CD8 ratio (Table 4). On the other hand, the TD DN T-cell frequency was positively correlated with CD8 T-cell counts and the duration of treatment and negatively with the CD4/CD8 ratio (Table 4), while the EM DN T-cell frequency was negatively correlated with both the duration of the infection and the treatment and positively with VL (Table 4). Frequencies of naïve (CD45RA^+^CD28^+^) DN T-cells remained stable despite HIV infection or early ART initiation (Figure 2A,F) vs. non-infected controls. Since the IL-7 receptor (CD127) is needed for T-cell survival and HIV infection is known to downregulate its expression by CD4 and CD8 T-cells [50,51,52], we also assessed CD127 expression by DN T-cells, which has not been evaluated in previous studies on DN T-cells during HIV/SIV infections. In this regard, the frequencies of CD127^+^ DN T-cells were lower in acute HIV infection compared to non-infected controls, and early ART initiation failed to restore their frequencies (Figure 2A,G). CD127^+^ DN T-cells were also positively correlated with the CD4/CD8 ratio (Table 4). Altogether, our findings confirmed previous reports of elevate frequencies of DN T-cells during HIV infection and showed that early ART initiation was unable to normalize their frequencies.

### 3.2. Dynamics of CD39- and CD73-Expressing DN T-Cells during Early HIV Infection and Following ART 

**Figure 3:**
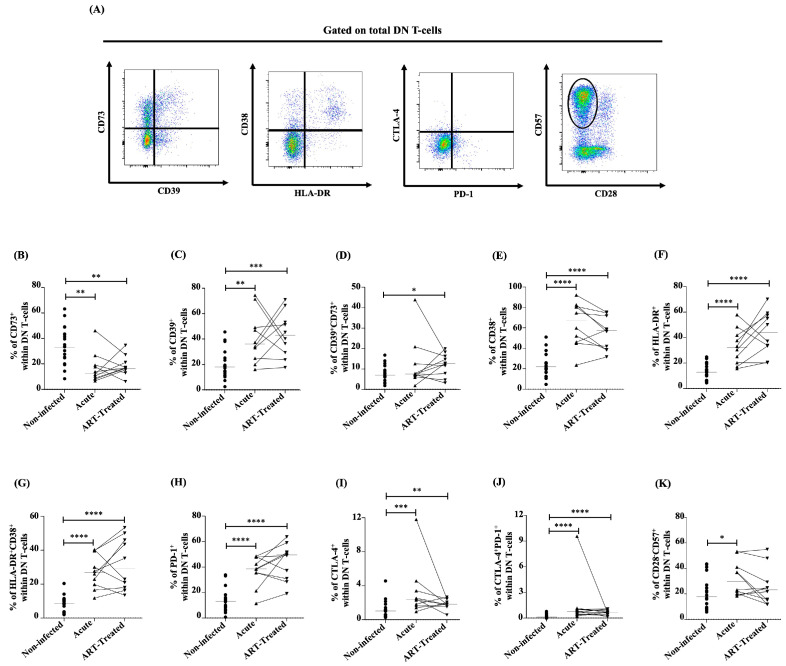
(**A**) Gating strategy used in flow cytometry to determine CD73/CD39, CD38/HLA-DR, CTLA-4/PD-1, and CD57/CD28 expression within DN T-cells in the human study. Percentages determined in flow cytometry of CD73^+^ (**B**), CD39^+^ (**C**), CD39^+^CD73^+^ (**D**), CD38^+^ (**E**), HLA-DR^+^ (**F**), HLA-DR^+^CD38^+^ (**G**), PD-1^+^ (**H**), CTLA-4^+^ (**I**), CTLA-4^+^PD-1^+^ (**J**), and CD28^−^CD57^+^ (**K**) within DN T-cells in the human study. After the Kruskal–Wallis analysis, the differences among the three study groups were determined by a nonparametric Mann–Whitney rank test for unpaired variables (non-infected vs. acute, non-infected vs. ART-treated) and Wilcoxon signed-rank test for paired variables (acute vs. ART-treated). Sample sizes in flow cytometry analysis: non-infected (*n* = 20), acute, and ART-treated (*n* = 10). Horizontal line s in graphs represent the median. Only statistical significances are presented (*, *p* < 0.05; **, *p* < 0.01; ***, *p* < 0.001; ****, *p* < 0.0001).

To evaluate the potential immunoregulatory roles of DN T-cells, we examined the expression of the ectonucleotidases CD39 and CD73. Indeed, CD39 converts inflammatory ATP into ADP and AMP, which is hydrolyzed by another ectonucleotidase, CD73, to produce immunosuppressive adenosine, inhibiting anti-HIV immune responses [53,54,55]. The frequencies of CD73^+^ DN T-cells were lower in the early phase of HIV infection and following early ART initiation compared to non-infected controls (Figure 3A,B), whereas CD39^+^ DN T-cell frequencies increased and remained higher than non-infected controls following ART initiation (Figure 3A,C). ART initiation was associated with increased CD39^+^CD73^+^ DN T-cell percentages compared to non-infected donors (Figure 3A,D). In contrast to CD39^+^ DN T-cells, the frequencies of CD73^+^ DN T-cells were negatively correlated with the CD8 T-cell count and positively with the CD4/CD8 ratio (Table 4). CD39^+^ DN T-cells were also positively correlated with the duration of the infection and duration of treatment (Table 4). Finally, CD39^+^CD73^+^ DN T-cells and the duration of the infection were positively correlated (Table 4). These results indicated that immunosuppressive CD39-expressing DN T-cells increased in the early phase of HIV infection and remained elevated despite ART.

### 3.3. Elevated Expression of Immune Activation, Immune Checkpoint, and Senescence Markers by DN T-Cells during Early HIV Infection Is Not Restored by ART Initiation 

We then analyzed the expression of cellular markers linked to CD4 and CD8 T-cells’ immune activation, exhaustion, and senescence, which we previously evaluated in DN T-cells [19]. First, PWH showed greater frequencies of activated DN T-cells as determined by CD38^+^ (Figure 3A,E) and HLA-DR^+^ (Figure 3A,F) than non-infected controls, which remained higher regardless of early ART initiation. Similarly, we observed higher frequencies of CD38^+^HLA-DR^+^ (Figure 3A,G) DN T-cells, which also remained high despite early treatment. Activated (CD38^+^, HLA-DR^+^, and CD38^+^HLA-DR^+^) DN T-cells were positively correlated with CD8 T-cell counts and negatively with the CD4/CD8 ratio, whereas CD38^+^ DN T-cells were also negatively correlated with CD4 T-cell counts (Table 4).

Secondly, we analyzed the expression of immune checkpoints PD-1 and CTLA-4, which were higher during early HIV infection and remained elevated compared to non-infected controls regardless of early ART initiation (Figure 3A,H–J). PD-1^+^ DN T-cells were positively correlated with CD8 T-cell counts and negatively with the CD4/CD8 ratio, and CD38^+^ DN T-cells were also negatively correlated with CD4 T-cell counts (Table 4). We then assessed senescent DN T-cells (CD28^−^CD57^+^), which were higher during early HIV infection and were restored by early ART initiation (Figure 3A,K) vs. non-infected donors. 

Senescent DN T-cells were negatively associated with CD4 T-cell counts and the CD4/CD8 ratio, while a positive association was observed with CD8 T-cell counts (Table 4). Overall, during acute HIV infection, DN T-cells showed increased levels of immune activation, immune checkpoint expression, and senescence markers, which, except for senescence markers, early ART initiation failed to restore.

### 3.4. Dynamics of DN Tregs during Early HIV Infection and Following ART 

**Figure 4:**
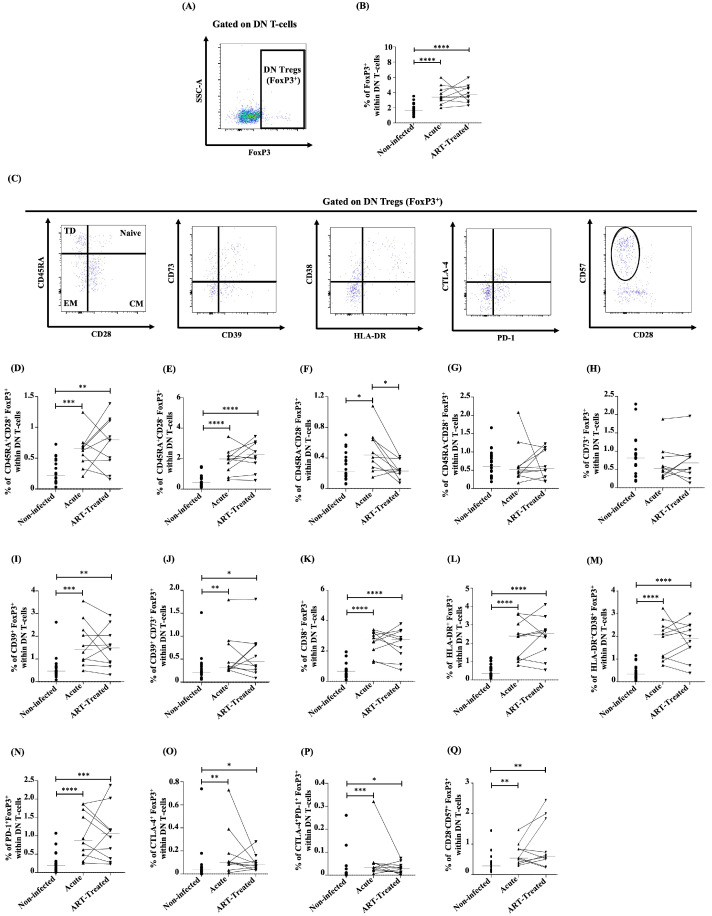
(**A**) Gating strategy used in flow cytometry to determine total FoxP3^+^ DN T-cells (DN Tregs) (left) in the human study. (**B**) Percentages determined in flow cytometry of total DN Tregs within DN T-cells in the human study. (**C**) Gating strategy used in flow cytometry to determine DN Treg memory subsets based on CD45RA/CD28, CD73/CD39, CD38/HLA-DR, CTLA-4/PD-1, and CD57/CD28 expression within DN Tregs in the human study. Percentages determined in flow cytometry of naïve (CD45RA^+^CD28^+^) (**D**), terminally differentiated (TD, CD45RA^+^CD28^−^) (**E**), effector memory (EM, CD45RA^−^CD28^−^) (**F**), central memory (CM, CD45RA^−^CD28^+^) (**G**), CD73^+^FoxP3^+^ (**H**), CD39^+^FoxP3^+^ (**I**), CD39^+^CD73^+^FoxP3^+^ (**J**), CD38^+^FoxP3^+^ (**K**), HLA-DR^+^FoxP3^+^ (**L**), HLA-DR^+^CD38^+^FoxP3^+^ (**M**), PD-1^+^FoxP3^+^ (**N**), CTLA-4^+^FoxP3^+^ (**O**), CTLA-4^+^PD-1^+^FoxP3^+^ (**P**), and CD28^−^CD57^+^FoxP3^+^ (**Q**) DN T-cells in the human study. After the Kruskal–Wallis analysis, the differences among the three study groups were determined by a nonparametric Mann–Whitney rank test for unpaired variables (non-infected vs. acute, non-infected vs. ART-treated) and Wilcoxon signed-rank test for paired variables (acute vs. ART-treated). Sample sizes in flow cytometry analysis: non-infected (*n* = 20), acute, and ART-treated (*n* = 10). Horizontal line in graphs represent the median. Only statistical significances are presented (*, *p* < 0.05; **, *p* < 0.01; ***, *p* < 0.001; ****, *p* < 0.0001).

We further explored the dynamics of DN T-cells expressing FoxP3 as the master transcription factor of regulatory T-cells (DN Tregs). The frequencies of total DN Tregs were higher in the early phase of the HIV infection vs. non-infected individuals, while ART failed to normalize their frequencies (Figure 4A,B). DN Tregs’ frequency was positively associated with CD8 T-cell counts and negatively with the CD4/CD8 ratio (Table 4). We then analyzed the phenotype of DN Tregs. We found that DN Tregs displayed increased naïve (CD45RA^+^CD28^+^) and terminally differentiated (TD, CD45RA^+^CD28^−^) phenotypes upon HIV infection, which were not normalized by early ART initiation (Figure 4C–E). The increase in effector memory (EM, CD45RA^−^CD28^−^) DN Tregs during acute HIV infection was normalized by early ART (Figure 4C,F), whereas no differences were found in CM (CD45RA^−^CD28^+^) DN Tregs after HIV infection and ART compared to uninfected controls (Figure 4C,G). Naïve and TD Tregs’ frequencies were positively correlated with CD8 T-cell counts and negatively with the CD4/CD8 ratio. In contrast, EM Tregs were positively associated with VL and negatively with the duration of the infection (Table 4). CM Tregs’ frequencies were negatively correlated with the duration of treatment (Table 4). Altogether, our findings confirmed previous reports of increased frequencies of DN Tregs during HIV infection and showed that early ART initiation was unable to normalize their frequencies.

Moreover, CD73^+^ DN Treg frequencies were similar in uninfected controls and PWH regardless of their ART status (Figure 4C,H). In contrast, CD39^+^ and CD39^+^CD73^+^ DN Tregs were higher during early HIV infection and continued to stay elevated after ART (Figure 4C,I,J) compared to non-infected controls. Similarly to CD39^+^ DN T-cell frequencies, CD39^+^ DN Tregs were positively correlated with CD8 T-cell counts and negatively with the CD4/CD8 ratio (Table 4). Finally, CD39^+^CD73^+^ DN Tregs were positively associated with CD8 T-cell counts (Table 4). These results indicated that immunosuppressive CD39-expressing DN Tregs were increased during early HIV infection and remained elevated despite ART.

The frequencies of CD38^+^ (Figure 4C,K), HLA-DR^+^ (Figure 4C,L), CD38^+^HLA-DR^+^ (Figure 4C,M), PD-1^+^ (Figure 4C,N), CTLA-4^+^ (Figure 4C,O), CTLA-4^+^PD-1^+^ (Figure 4C,P), and (CD28^−^CD57^+^) (Figure 4C,Q) DN Tregs were augmented during early HIV infection, and ART was unable to normalize their frequencies to the levels seen in non-infected controls. Activated (CD38^+^, HLA-DR^+^, and CD38^+^HLA-DR^+^) and PD-1^+^ DN Tregs were positively correlated with CD8 T-cell counts and negatively with the CD4/CD8 ratio (Table 4). Similarly, senescent DN Tregs were negatively associated with CD4 T-cell counts and the CD4/CD8 ratio, while a positive correlation was observed with CD8 T-cell counts (Table 4). Overall, during acute HIV infection, DN Tregs showed increased levels of immune activation, immune checkpoint expression, and senescence markers, which early ART initiation failed to restore.

### 3.5. Increased Expression of Chemokine Receptors by DN T-Cells and DN Tregs during Acute HIV Infection Was Not Normalized by Early ART Initiation

**Figure 5:**
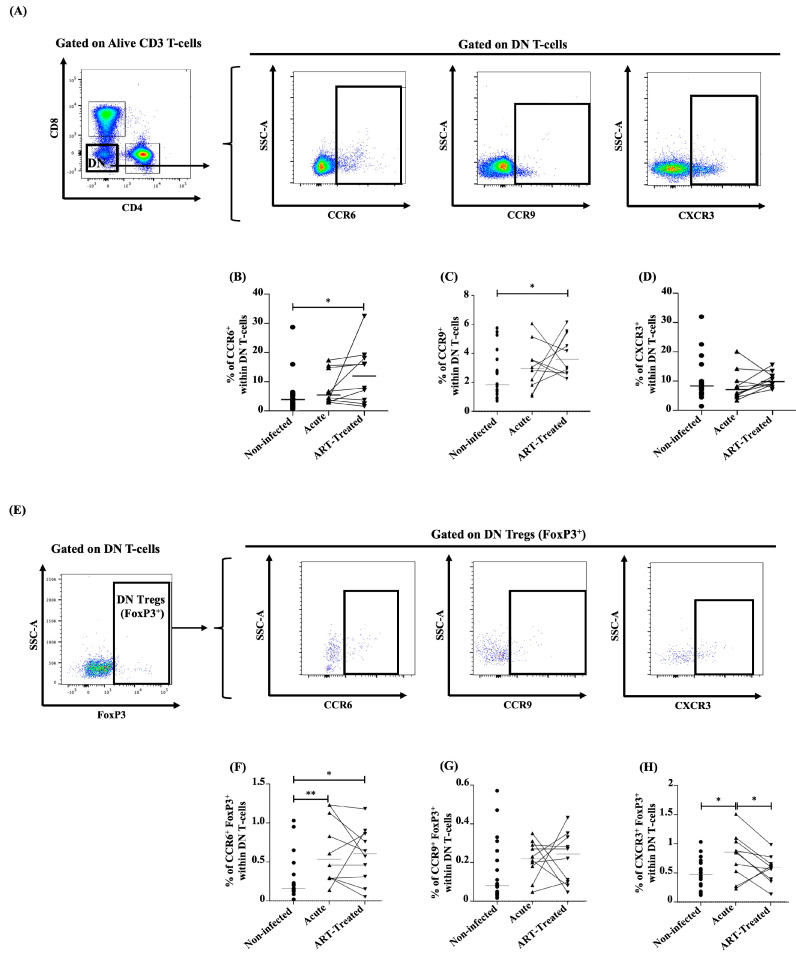
(**A**) Gating strategy used in flow cytometry to determine CCR6, CCR9, and CXCR3 expression within DN T-cells in the human study. Percentages determined in flow cytometry of CCR6^+^ (**B**), CCR9^+^ (**C**), and CXCR3^+^ (**D**) within DN T-cells in the human study. (**E**) Gating strategy used in flow cytometry to determine CCR6, CCR9, and CXCR3 expression within DN Tregs in the human study. Percentages determined in flow cytometry of CCR6^+^FoxP3^+^ (**F**), CCR9^+^FoxP3^+^ (**G**), and CXCR3^+^FoxP3^+^ (**H**) within DN T-cells in the human study. After the Kruskal–Wallis analysis, the differences among the three study groups were determined by a nonparametric Mann–Whitney rank test for unpaired variables (non-infected vs. acute, non-infected vs. ART-treated) and Wilcoxon signed-rank test for paired variables (acute vs. ART-treated). Sample sizes in flow cytometry analysis: non-infected (*n* = 20), acute, and ART-treated (*n* = 10). Horizontal lines in graphs represent the median. Only statistical significances are presented (*, *p* < 0.05; **, *p* < 0.01).

Frequencies of chemokine receptor CCR6^+^, a marker of T-cell homing towards inflammatory sites and the gut (Figure 5A,B), and CCR9^+^, a gut-homing marker (Figure 5A,C), DN T-cells remained unchanged during acute HIV infection while their frequencies were higher in early ART-treated individuals (Figure 5A–C). The frequencies of CXCR3^+^ DN T-cells remained similar to those observed in non-infected individuals despite HIV infection and early ART initiation (Figure 5A,D). Interestingly, similar dynamics of CXCR3 and CCR9 expression were observed in CD4^+^ and CD8^+^ T-cells, whereas decreased CCR6 expression by both CD4^+^ and CD8^+^ T-cells was observed in ART-treated individuals compared to those found in DN T-cells (data not shown). Furthermore, CCR6^+^ DN Treg frequencies were higher in acute HIV infection vs. non-infected controls, and early ART initiation failed to restore their frequencies (Figure 5E,F), whereas frequencies of CCR9^+^ DN Tregs were similar in non-infected controls and PWH (Figure 5E,G). Expression of CXCR3^+^, a marker of migration to inflammatory sites by DN Tregs, was elevated during acute HIV infection, and early ART initiation normalized their frequencies (Figure 5E,H). Altogether, these results suggest that during acute HIV infection, DN T-cells show increased homing marker expression towards inflammatory sites and the gut, and this increase is not reversed by early ART initiation.

### 3.6. Dynamics of DN T-Cells and DN Tregs during Acute SIV Infection and Following Very Early ART Initiation

**Figure 6:**
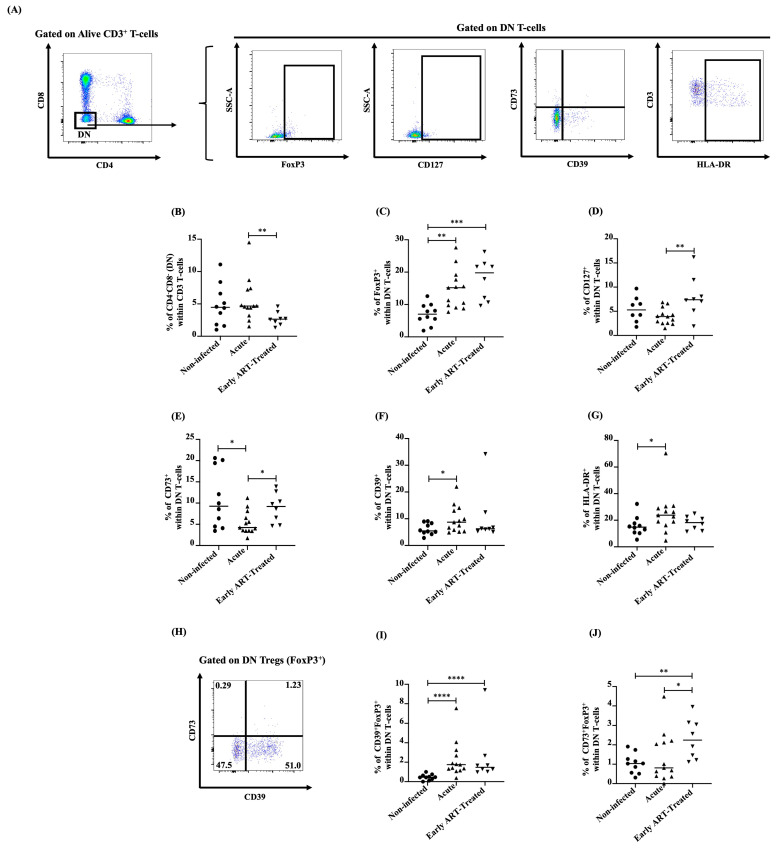
(**A**) Gating strategy used in flow cytometry to determine total CD3^+^CD4^−^CD8^−^ (double-negative, DN) T-cells, total FoxP3^+^ DN T-cells (DN Tregs), CD127^+^, CD37/CD73, and HLA-DR^+^ DN T-cells in Rhesus macaques. Percentages determined in flow cytometry of total DN T-cells (**B**), total DN Tregs (**C**), CD127^+^ (**D**), CD73^+^ (**E**), CD39^+^ (**F**), and HLA-DR^+^ (**G**) within DN T-cells in Rhesus macaques. (**H**) Gating strategy used in flow cytometry to determine CD39/CD73 within DN Tregs in Rhesus macaques. Percentages determined in flow cytometry of CD39^+^FoxP3^+^ (**I**) and CD73^+^FoxP3^+^ (**J**) within DN T-cells in Rhesus macaques. After the Kruskal–Wallis analysis, the differences among the three study groups were determined by a nonparametric Mann–Whitney rank test for unpaired variables (non-infected vs. acute, non-infected vs. early ART-treated, and acute vs. early ART-treated) in Rhesus macaques. Sample sizes in flow cytometry analysis in Rhesus macaques: non-infected (*n* = 10), acute (*n* = 13), and early ART-treated (*n* = 8). Horizontal lines in graphs represent the median. Only statistical significances are presented (*, *p* < 0.05; **, *p* < 0.01; ***, *p* < 0.001; ****, *p* < 0.0001).

To evaluate the effect of very early ART initiation on DN and DN Tregs’ dynamics, we used an acute SIV infection model of female RMs in which ART was initiated at four days post-infection (Figure 1). Here, total DN T-cells remained unchanged during acute SIV infection, and very early ART initiation significantly decreased their number compared to the acutely infected group (Figure 6A,B). DN T-cells were positively correlated with VL (Table 5). However, similar to the human study, DN Tregs’ frequencies increased during acute SIV infection, and very early ART initiation was unable to normalize them (Figure 6A,C). CD127^+^ DN T-cells tended to decrease in acute SIV-infected animals without reaching statistical significance, while very early ART initiation increased their frequencies compared to monkeys in the acute phase of the infection (Figure 6A,D). CD127^+^ DN T-cells were negatively correlated with VL (Table 5). In contrast to CD73^+^ DN T-cells, which decreased in acute SIV infection and whose frequencies were normalized by very early ART initiation (Figure 6A,E), CD39^+^ DN T-cells increased during acute SIV infection, and very early ART initiation restored their frequencies (Figure 6A,F). In addition, activated DN T-cells’ (HLA-DR^+^ DN) percentages increased during acute SIV infection, and very early initiation normalized their levels (Figure 6A,G). Moreover, CD39^+^ DN Treg frequencies are higher in SIV-infected RMs regardless of their ART status than in non-infected animals (Figure 6H,I). In contrast, CD73^+^ DN Tregs decreased during the acute phase of the infection, and very early ART initiation increased their frequencies (Figure 6H,J). CD39^+^ DN T-cells were negatively correlated with CD4 T-cell counts and the CD4/CD8 ratio and positively associated with the duration of the infection, whereas CD39^+^ DN Tregs were negatively associated with the CD4/CD8 ratio (Table 5). Our RM model showed that in contrast to the observations in the human study, the frequencies of DN T-cells and CD127^+^ DN T-cells remained stable during acute SIV infection. However, the dynamics of DN Tregs, activated DN T-cells, and CD73/CD39-expressing DN T-cells and DN Tregs were similar in acutely SIV-infected RM and acute HIV-infected individuals.

## 4. Discussion

Contradictory reports on the dynamics of DN T-cells during HIV infection have been published, including those showing an increase in DN T-cell frequencies [39] and others showing their decreased numbers [40,41,42]. Early ART initiation as close as possible to the estimated time of HIV exposure is strongly recommended in clinical practice due to its demonstrated benefits in promoting CD4 T-cell count recovery, reducing immune activation, and lowering the risk of secondary HIV transmission [56,57]. In our study, early ART initiation in the human study was assessed in the first six months of the infection, whereas very early ART initiation was evaluated in the RM study at four days post-infection.

Overall, our results indicate that acute SIV infection recapitulated the observations in acute HIV-infected individuals for most of the analyzed parameters (i.e., frequencies of DN Tregs, CD39/CD73 expression, and HLA-DR expression) except for the dynamics of total DN T-cells and CD127 expression. At a first look, the observed increases in the frequencies of total DN T-cells during the acute HIV infection contrast with a previous report of reduced DN T-cell counts in individuals with high VL during acute infection [41]. However, in their manuscript, individuals with CD4 > 500 and log10 VL > 4.5 have higher DN T-cell counts than uninfected individuals. In contrast, in our study, the median of CD4 T-cell counts was 450 cells/μL, and the median of log10 VL was 4.4, corresponding to DN T-cell counts higher in PWH compared to uninfected individuals in their study. 

On the other hand, the maintenance of DN T-cell frequencies seems to be an intrinsic characteristic of the pathogenic SIV infection in RMs, as previously reported [43] and consistent with our observations. In our human cohort, we observed decreased CD127 expression in acutely infected participants that was not restored by early ART initiation, which is in line with reports of CD127 downregulation in both CD4 and CD8 T-cells during HIV infection [50,51,52]. Moreover, CD127 expression remained stable in SIV-infected RMs, and its frequency increased upon very early ART initiation. This could suggest that CD127 downregulation might occur slower in RMs compared to PWH and that very early ART initiation can be beneficial for the recovery of its expression. CD127 downregulation may indicate dysregulation in cell survival and homeostasis and aligns with higher senescence, exhaustion, and potential dysfunction in DN T-cells [58].

A distinctive differentiation pattern of DN T-cell subsets was observed during acute HIV infection, characterized by stable frequencies of naïve and EM subsets along with decreased CM and increased TD DN T-cells that were not normalized by early ART. Indeed, the increase in TD DN T-cells and their persistence despite early ART initiation are consistent with the increased frequencies of activated (CD38^+^, HLA-DR^+^, CD38^+^HLA-DR^+^), immunosenescent (CD28^−^CD57^+^), and PD-1/CTLA-4-expressing DN T-cells, indicating that these cells exhibit exhausted features and might have impaired functions. These observations align with the increased immune activation, exhaustion, and senescence observed in CD4 and CD8 T-cells within the same study cohort, as we previously reported [59]. EM T-cells exhibit an enhanced capacity to localize within tissues and migrate into non-lymphoid areas in response to infection or inflammation [60,61], suggesting that during acute HIV infection, their migratory potential towards inflammatory sites and the gut remained unaffected, which is in line with the observed stability in CCR6, CCR9, and CXCR3 expression during acute HIV infection. In contrast, EM DN Tregs were increased during acute HIV infection in accordance with higher CCR6, CCR9, and CXCR3 expression in PWH compared to uninfected participants, which could contribute to disease progression and mucosal fibrosis. We previously reported similar results in the same study cohort for CD4^+^ Tregs [59].

In both acute HIV and SIV infections, the frequencies of DN Tregs increased and were not restored by early or even very early ART initiation. In the human cohort, frequencies of DN Tregs positively correlated with CD8 T-cell counts and inversely with the CD4/CD8 ratio, consistent with a previous report of DN Treg accumulation during HIV infection and its association with disease progression and impaired immune responses [47]. In this sense, DN Tregs expressed higher levels of CD39, HLA-DR/CD38, and CTLA-4/PD-1, which are all necessary for CD4^+^ Tregs’ immunosuppressive functions and proliferation [62,63,64,65,66,67,68,69]; therefore, we cannot exclude the possibility that similar mechanisms might occur for DN Tregs. The increase in total DN Tregs and their subsets, along with the persistence of elevated frequencies despite early ART initiation, could be associated with persistent immune activation in PWH since immune activation induces Treg differentiation [70], and we previously reported persistent immune activation in the same study cohort [59]. 

Increased CD39 expression is documented to be associated with HIV disease progression and chronic immune dysfunction [68,69], whereas decreased CD73 expression is associated with T-cell immune activation and exhaustion in HIV infection [71]. Here, we observed increased CD39 expression by both DN T-cells and DN Tregs during acute HIV and SIV infections, and only CD39^+^ DN T-cells were normalized after very early ART initiation in SIV-infected RM. These results indicate that the timing of ART initiation is important to control CD39 expression by total DN T-cells but has no effect on controlling CD39 expression in DN Tregs, similar to our previous findings on CD39^+^ CD4^+^ Tregs in the same study cohort [59]. Furthermore, we only observed a decrease in CD73 expression in total DN T-cells during acute HIV infection, which was not normalized by early ART initiation. The reduction in CD73^+^ DN T-cells aligns with a previous report in which the decrease in these cells was correlated with and predicted poor immune reconstitution in PWH [72]. Nevertheless, the authors of the above publication found a normalization of CD73 expression by DN T-cells with long-term ART (median of 5.7 years), which contrasts with the ART duration in our study cohort (median 1.72 years), indicating that a longer ART duration might normalize the frequencies of this subset.

Moreover, the frequencies of various DN T-cell subsets were associated with clinical parameters usually linked to disease progression. As such, activated DN T-cells, PD-1^+^ DN T-cells, DN Tregs, CD39^+^ DN Tregs, activated DN Tregs, and PD-1^+^ DN Tregs positively correlated with CD8 T-cell counts and negatively with the CD4/CD8 ratio. In addition, CD39^+^ DN T-cells were positively associated with both the duration of infection and treatment and negatively correlated with the CD4/CD8 ratio, which aligns with previous reports connecting CD39 expression and HIV disease progression [54,69]. The lack of correlations between DN T-cell subsets and clinical or immunological parameters in the RM study may be attributed to the differential dynamics of CD8 T-cells following SIV infection and the very early ART initiation in this model compared to HIV infection and ART in humans. Indeed, CD8 T-cell counts are normalized following ART in humans, but their number increased upon very early ART initiation in RM.

One of the limits of our study is the relatively small sample size, which may impact the statistical power of our results. Replication studies with larger cohorts would be beneficial in confirming our findings. Due to limited specimen availability, we were unable to perform in vitro functional assays, which could have provided additional insights into DN T-cells’ and DN Tregs’ functions. Furthermore, no markers to assess memory subsets were included in the RM study. Moreover, while we used established markers for T-cell migration to gut and inflammatory sites, all assessments were conducted using peripheral blood samples to indicate DN T-cell migration patterns indirectly. To validate our observations, further investigations should focus on evaluating DN T-cell dynamics directly in gut mucosal tissue rather than relying solely on peripheral blood markers. In addition, assessing viral reservoirs in different DN T-cells and DN Treg subsets will be the focus of upcoming publications. However, despite the limitations, our findings align with our previous research outcomes and demonstrate high biological plausibility. 

## 5. Conclusions

This study revealed that acute HIV infection significantly alters the frequencies and functional properties of DN T-cells and DN Tregs, with these alterations not fully restored by early ART initiation. Similar patterns were observed in SIV-infected RM, where very early ART initiation normalized some but not all DN T-cell and DN Treg populations. These findings highlight the persistent immune dysregulation despite early ART initiation and underscore the need for additional therapeutic strategies targeting DN T-cells to achieve full immune recovery in PWH.

## Figures and Tables

**Table 1:** Clinical characteristics of study groups in the human study.

Characteristics	Study Population (*n* = 30)		
	Non-Infected (*n* = 20)	Acute (*n* = 10)	ART-Treated (*n* = 10)
Male sex, *n* (%)	15 (75%)	10 (100%)	10 (100%)
Age, years [median (IQR)]	39 [30.75–47]	36 [29.75–46.5]	36.5 [29–46.5]
CD4^+^ T-cell count, cells/µL [median (IQR)]	632 ^a^ [463.5–775]	450 ^a,c^ [272.5–561.3]	521 ^c^ [377.5–795]
CD8^+^ T-cell count, cells/µL [median (IQR)]	197 ^a,b^ [153–428.5]	1019 ^a^ [580–1708]	655 ^b^ [531–1081]
CD4^+^/CD8^+^ ratio [median (IQR)]	2.82 ^a,b^ [1.41–4.19]	0.40 ^a,c^ [0.19–0.81]	0.69 ^b,c^ [0.40–1.24]
Viral load, log_10_ copies/mL [median (IQR)]	N/A	4.40 ^c^ [3.93–5.77]	1.70 ^c^ [1.68–1.70]
Duration of infection, months [median (IQR)]	N/A	3.36 ^c^ [1.56–4.68]	27.24 ^c^ [24–29.52]
Time of ART initiation post-infection, months [median (IQR)]	N/A	N/A	5.52 [3.24–7.08]
Duration of ART, months [median (IQR)]	N/A	N/A	20.64 [17.16–24]

Significant differences (*p* < 0.05) following Mann–Whitney U test, Fisher’s test, and Wilcoxon signed-rank test are mentioned as follows: a: non-infected vs. acute, b: non-infected vs. ART-treated, c: acute vs. ART-treated. N/A: non-applicable.

**Table 2:** Clinical characteristics of study groups in the Rhesus macaque study.

Characteristics	Study Population (*n* = 31)		
	Non-Infected (*n* = 10)	Acute (*n* = 13)	Early ART-Treated (*n* = 8)
CD4 count/mm^3^ [median (IQR)]	1056 ^a^ [766.3–1871]	673 ^a,c^ [513–980.5]	1472 ^c^ [1020–2687]
CD8 count/mm^3^ [median (IQR)]	648 [303–1231]	724 ^c^ [463.5–946.5]	1330 ^c^ [816–1986]
CD4^+^/CD8^+^ ratio [median (IQR)]	1.74 ^a,b^ [1.37–2.16]	0.98 ^a^ [0.67–1.57]	1.25 ^b^ [1.07–1.33]
Plasma viral load log_10_ copies/mL [median (IQR)]	N/A	6.76 ^c^ [6.12–7.39]	1.60 ^c^ [1.60–1.60]
Duration of infection (days) [median (IQR)]	N/A	33 [14–36]	27.5 [14–35]
Duration of ART (days) [median (IQR)]	N/A	N/A	23.5 [10–31]

Significant differences (*p* < 0.05) following Mann–Whitney U test are mentioned as follow: a: non-infected vs. acute, b: non-infected vs. early ART-treated, c: acute vs. early ART-treated. N/A: non-applicable.

**Table 3:** List of antibodies used in the flow cytometry analysis.

mAb	Fluorochrome	Clone	Company	Catalog
CD127	PE-Cy7	HIL-7R-M21	BD Pharmingen™	560822
CD152 (CTLA-4)	APC	BNI3	BD Pharmingen™	555855
CD183 (CXCR3)	PE-Cy5	1C6/CXCR3	BD Pharmingen™	551128
CD194 (CCR4)	BV421	1G1	BD Horizon™	562579
CD195 (CCR5)	BV605	2D7/CCR5	BD Horizon™	563379
CD196 (CCR6)	BB515	11A9	BD Horizon™	564479
CD199 (CCR9)	APC	L053E8	BioLegend	358908
CD279 (PD-1)	BV711	EH12.2H7	BioLegend	329928
CD28	PE-Cy5	CD28.2	BD Pharmingen™	555730
CD3	Alexa Fluor 700	UCHT1	BD Pharmingen™	557943
CD3	BV605	UCHT1	BD OptiBuild™	742623
CD3	BV786	UCHT1	BD Horizon™	565491
CD3	Alexa Fluor 488	SP34.2	BD Pharmingen™	557705
CD3	Alexa Fluor 700	SP34.2	BD Pharmingen™	557917
CD3	Alexa Fluor 488	SP34.2	BD Pharmingen™	557705
CD38	PE-Cy7	HIT2	BD Pharmingen™	560677
CD39	APC	eBioA1	eBioscience™	17-0399-42
CD39	BV711	TU66	BD Horizon™	563680
CD4	APC-H7	RPA-T4	BD Pharmingen™	560158
CD4	FITC	RPA-T4	BD Pharmingen™	555346
CD4	BV650	L200	BD Horizon™	563737
CD45RA	BV650	HI100	BD Horizon™	563963
CD45RA	APC-H7	5H9	BD Pharmingen™	561212
CD57	BV421	NK-1	BD Horizon™	568894
CD73	BV605	AD2	BD Horizon™	563199
CD8α	APC-H7	SK1	BD Pharmingen™	560179
CD8α	APC-R-700	SK1	BD Horizon™	565192
CD8β	PE-Cy7	SIDI8BEE	eBioscience™	25-5273-42
FoxP3	PE-CF594	236/A7	BD Horizon™	563955
HLA-DR	BV605	G46-6	BD Pharmingen™	562844
Integrin β7	FITC	FIB504	BioLegend	321212
LAP(TGF-β1)	BV421	TW4-2F8	BioLegend	349613

**Table 4:** Correlation between HIV clinical and virological parameters and percentages determined by flow cytometry of subsets of DN T-cells and DN Tregs in the human study.

Correlations in Human Study						
	CD4 T-Cell Count	CD8 T-Cell Count	CD4/CD8 Ratio	log_10_ VL	Duration of Infection	Duration of Treatment
Total DN T-cells	N.S.	*p =* 0.03; r = 0.39	N.S.	N.S.	N.S.	N.S.
Central memory (CM, CD45RA^−^CD28^+^) DN T-cells	N.S.	*p =* 0.003; r = −0.53	*p =* 0.001; r = 0.56	N.S.	N.S.	*p =* 0.03; r = −0.68
Effector memory (EM, CD45RA^−^CD28^−^) DN T-cells	N.S.	N.S.	N.S.	*p =* 0.004; r = 0.61	*p =* 0.0007; r = −0.69	*p =* 0.01; r = −0.75
Terminally differentiated (TD, CD45RA^−^CD28^+^) DN T-cells	N.S.	*p =* 0.009; r = 0.47	*p =* 0.005; r = −0.50	N.S.	N.S.	*p =* 0.01; r = 0.77
FoxP3^+^ DN T-cells (DN Tregs)	N.S.	*p =* 0.007; r = 0.49	*p =* 0.02; r = −0.42	N.S.	N.S.	N.S.
Naïve DN Tregs (CD45RA^+^CD28^+^FoxP3^+^)	N.S.	*p =* 0.003; r = 0.53	*p =* 0.007; r = −0.49	N.S.	N.S.	N.S.
Central memory DN Tregs (CM, CD45RA^−^CD28^+^FoxP3^+^)	N.S.	N.S.	N.S.	N.S.	N.S.	*p =* 0.004; r = −0.83
Effector memory DN Tregs (EM, CD45RA^−^CD28^−^FoxP3^+^)	N.S.	N.S.	N.S.	*p =* 0.007; r = 0.58	*p =* 0.001; r = −0.66	N.S.
Terminally differentiated DN Tregs (TD, CD45RA^−^CD28^+^FoxP3^+^)	N.S.	*p =* 0.004; r = 0.51	*p =* 0.01; r = −0.45	N.S.	N.S.	N.S.
CD127^+^ DN T-cells	N.S.	N.S.	*p =* 0.01; r = 0.46	N.S.	N.S.	N.S.
CD73^+^ DN T-cells	N.S.	*p =* 0.006; r = −0.50	*p =* 0.002; r = 0.53	N.S.	N.S.	N.S.
CD39^+^ DN T-cells	N.S.	*p =* 0.01; r = 0.44	*p =* 0.02; r = −0.42	N.S.	*p =* 0.05; r = 0.44	*p =* 0.02; r = 0.73
CD39^+^CD73^+^ DN T-cells	N.S.	N.S.	N.S.	N.S.	*p =* 0.04; r = 0.46	N.S.
CD39^+^ DN Tregs	N.S.	*p =* 0.01; r = 0.44	*p =* 0.03; r = −0.39	N.S.	N.S.	N.S.
CD39^+^CD73^+^ DN Tregs	N.S.	*p =* 0.05; r = 0.36	N.S.	N.S.	N.S.	N.S.
CD38^+^ DN T-cells	*p* = 0.01; r = −0.45	*p* < 0.0001; r = 0.70	*p* < 0.0001; r = −0.74	N.S.	N.S.	N.S.
HLA-DR^+^ DN T-cells	N.S.	*p =* 0.005; r = 0.50	*p =* 0.01; r = −0.44	N.S.	*p =* 0.05; r = 0.44	N.S.
HLA-DR^+^CD38^+^ DN T-cells	N.S.	*p =* 0.003; r = 0.52	N.S.	N.S.	N.S.	N.S.
PD-1^+^ DN T-cells	N.S.	*p =* 0.01; r = 0.46	*p =* 0.02; r = −0.42	N.S.	*p =* 0.01; r = 0.54	*p =* 0.04; r = 0.65
CTLA-4^+^PD-1^+^ DN T-cells	N.S.	*p =* 0.04; r = 0.37	*p =* 0.05; r = −0.36	N.S.	N.S.	N.S.
Senescent (CD28^−^CD57^+^) DN T-cells	*p =* 0.0003; r = −0.62	*p =* 0.01; r = 0.46	*p =* 0.0005; r = −0.61	N.S.	N.S.	N.S.
CD38^+^ DN Tregs	N.S.	*p =* 0.0006; r = 0.60	*p =* 0.001; r = −0.57	N.S.	N.S.	N.S.
HLA-DR^+^ DN Tregs	N.S.	*p =* 0.004; r = 0.52	*p =* 0.01; r = −0.47	N.S.	N.S.	N.S.
HLA-DR^+^CD38^+^ DN Tregs	N.S.	*p =* 0.001; r = 0.55	N.S.	N.S.	N.S.	N.S.
PD-1^+^ DN Tregs	N.S.	*p =* 0.03; r = 0.40	*p =* 0.04; r = −0.38	N.S.	N.S.	N.S.
CTLA-4^+^PD-1^+^ DN Tregs	*p =* 0.05; r = −0.37	N.S.	N.S.	N.S.	N.S.	N.S.
Senescent (CD28^−^CD57^+^) DN Tregs	*p =* 0.02; r = −0.41	*p =* 0.006;r = 0.49	*p =* 0.004;r = −0.52	N.S.	N.S.	N.S.

*p*-values are from comparing clinical and virological parameters with flow cytometry measurements by using the Spearman correlation coefficient test. N.S.: non-significant.

**Table 5:** Correlation between SIV clinical and virological parameters and percentages determined by flow cytometry of subsets of DN T-cells and DN Tregs in the Rhesus macaque study.

Correlations in Rhesus Macaque Study						
	CD4 T-Cell Count	CD8 T-Cell Count	CD4/CD8 Ratio	log_10_VL	Duration of Infection	Duration of Treatment
Total DN T-cells	N.S.	N.S.	N.S.	*p* = 0.0003; r = 0.71	N.S.	N.S.
CD127^+^ DN T-cells	N.S.	N.S.	N.S.	*p* = 0.02; r = −0.60	N.S.	N.S.
CD39^+^ DN T-cells	*p* = 0.05; r = −0.34	N.S.	*p* = 0.005; r = −0.49	N.S.	*p* = 0.05; r = 0.43	N.S.
CD39^+^ DN Tregs	N.S.	N.S.	*p* = 0.0004; r = −0.60	N.S.	N.S.	N.S.

*p*-values are from comparing clinical and virological parameters with flow cytometry measurements by using the Spearman correlation coefficient test. N.S.: non-significant.

## Data Availability

The data presented in this manuscript can be obtained from the corresponding author upon request, subject to reasonable conditions.

## Supplementary Materials

The following supporting information can be downloaded at https://www.mdpi.com/article/10.3390/v16101609/s1, Table S1: Description of viral load in all human study participants; Table S2: Description of viral load in Rhesus macaques.

## References

[1] Wu Z., Zheng Y., Sheng J., Han Y., Yang Y., Pan H., Yao J. (2022). CD3(+)CD4(-)CD8(-) (Double-Negative) T Cells in Inflammation, Immune Disorders and Cancer. Front. Immunol..

[2] Velikkakam T., Gollob K.J., Dutra W.O. (2022). Double-negative T cells: Setting the stage for disease control or progression. Immunology.

[3] Bafor E.E., Valencia J.C., Young H.A. (2022). Double Negative T Regulatory Cells: An Emerging Paradigm Shift in Reproductive Immune Tolerance?. Front. Immunol..

[4] Li H., Tsokos G.C. (2021). Double-negative T cells in autoimmune diseases. Curr. Opin. Rheumatol..

[5] Mixter P.F., Russell J.Q., Morrissette G.J., Charland C., Aleman-Hoey D., Budd R.C. (1999). A model for the origin of TCR-alphabeta+ CD4-CD8- B220+ cells based on high affinity TCR signals. J. Immunol..

[6] Pobezinsky L.A., Angelov G.S., Tai X., Jeurling S., Van Laethem F., Feigenbaum L., Park J.H., Singer A. (2012). Clonal deletion and the fate of autoreactive thymocytes that survive negative selection. Nat. Immunol..

[7] Rodríguez-Rodríguez N., Apostolidis S.A., Penaloza-MacMaster P., Martín Villa J.M., Barouch D.H., Tsokos G.C., Crispín J.C. (2015). Programmed cell death 1 and Helios distinguish TCR-αβ+ double-negative (CD4-CD8-) T cells that derive from self-reactive CD8 T cells. J. Immunol..

[8] Pham T.N., Lukhele S., Hajjar F., Routy J.P., Cohen E.A. (2014). HIV Nef and Vpu protect HIV-infected CD4+ T cells from antibody-mediated cell lysis through down-modulation of CD4 and BST2. Retrovirology.

[9] Rodriguez-Rodriguez N., Flores-Mendoza G., Apostolidis S.A., Rosetti F., Tsokos G.C., Crispin J.C. (2020). TCR-alpha/beta CD4(-) CD8(-) double negative T cells arise from CD8(+) T cells. J. Leukoc. Biol..

[10] Apetrei C., Gaufin T., Gautam R., Vinton C., Hirsch V., Lewis M., Brenchley J., Pandrea I. (2010). Pattern of SIVagm infection in patas monkeys suggests that host adaptation to simian immunodeficiency virus infection may result in resistance to infection and virus extinction. J. Infect. Dis..

[11] Zlotnik A., Godfrey D.I., Fischer M., Suda T. (1992). Cytokine production by mature and immature CD4-CD8- T cells. Alpha beta-T cell receptor+ CD4-CD8- T cells produce IL-4. J. Immunol..

[12] Alunno A., Carubbi F., Bistoni O., Caterbi S., Bartoloni E., Bigerna B., Pacini R., Beghelli D., Cipriani P., Giacomelli R. (2014). CD4(-)CD8(-) T-cells in primary Sjögren's syndrome: Association with the extent of glandular involvement. J. Autoimmun..

[13] Poddighe D., Dossybayeva K., Kozhakhmetov S., Rozenson R., Assylbekova M. (2024). Double-Negative T (DNT) Cells in Patients with Systemic Lupus Erythematosus. Biomedicines.

[14] Petitjean G., Chevalier M.F., Tibaoui F., Didier C., Manea M.E., Liovat A.S., Campa P., Müller-Trutwin M., Girard P.M., Meyer L. (2012). Level of double negative T cells, which produce TGF-β and IL-10, predicts CD8 T-cell activation in primary HIV-1 infection. Aids.

[15] Bhatnagar N., Girard P.M., Lopez-Gonzalez M., Didier C., Collias L., Jung C., Bollens D., Duvivier C., Von Platen C., Scott-Algara D. (2017). Potential Role of Vδ2(+) γδ T Cells in Regulation of Immune Activation in Primary HIV Infection. Front. Immunol..

[16] Ohga S., Nomura A., Takahata Y., Ihara K., Takada H., Wakiguchi H., Kudo Y., Hara T. (2002). Dominant expression of interleukin 10 but not interferon gamma in CD4(-)CD8(-)alphabetaT cells of autoimmune lymphoproliferative syndrome. Br. J. Haematol..

[17] Fang L., Ly D., Wang S.S., Lee J.B., Kang H., Xu H., Yao J., Tsao M.S., Liu W., Zhang L. (2019). Targeting late-stage non-small cell lung cancer with a combination of DNT cellular therapy and PD-1 checkpoint blockade. J. Exp. Clin. Cancer Res..

[18] Merims S., Li X., Joe B., Dokouhaki P., Han M., Childs R.W., Wang Z.Y., Gupta V., Minden M.D., Zhang L. (2011). Anti-leukemia effect of ex vivo expanded DNT cells from AML patients: A potential novel autologous T-cell adoptive immunotherapy. Leukemia.

[19] Meziane O., Salahuddin S., Pham T.N.Q., Farnos O., Pagliuzza A., Olivenstein R., Thomson E., Alexandrova Y., Orlova M., Schurr E. (2020). HIV Infection and Persistence in Pulmonary Mucosal Double Negative T Cells In Vivo. J. Virol..

[20] Zhang Z.-X., Ma Y., Wang H., Arp J., Jiang J., Huang X., He K.M., Garcia B., Madrenas J.m., Zhong R. (2006). Double-Negative T Cells, Activated by Xenoantigen, Lyse Autologous B and T Cells Using a Perforin/Granzyme-Dependent, Fas-Fas Ligand-Independent Pathway. J. Immunol..

[21] Khan N., Geiger J.D. (2021). Role of Viral Protein U (Vpu) in HIV-1 Infection and Pathogenesis. Viruses.

[22] Lindwasser O.W., Chaudhuri R., Bonifacino J.S. (2007). Mechanisms of CD4 downregulation by the Nef and Vpu proteins of primate immunodeficiency viruses. Curr. Mol. Med..

[23] Chen B.K., Gandhi R.T., Baltimore D. (1996). CD4 down-modulation during infection of human T cells with human immunodeficiency virus type 1 involves independent activities of vpu, env, and nef. J. Virol..

[24] Willey R.L., Maldarelli F., Martin M.A., Strebel K. (1992). Human immunodeficiency virus type 1 Vpu protein regulates the formation of intracellular gp160-CD4 complexes. J. Virol..

[25] El-Far M., Ancuta P., Routy J.P., Zhang Y., Bakeman W., Bordi R., DaFonseca S., Said E.A., Gosselin A., Tep T.S. (2015). Nef promotes evasion of human immunodeficiency virus type 1-infected cells from the CTLA-4-mediated inhibition of T-cell activation. J. Gen. Virol..

[26] Crise B., Buonocore L., Rose J.K. (1990). CD4 is retained in the endoplasmic reticulum by the human immunodeficiency virus type 1 glycoprotein precursor. J. Virol..

[27] Rahmberg A.R., Markowitz T.E., Mudd J.C., Hirsch V., Brenchley J.M. (2022). Epigenetic Reprogramming Leads to Downregulation of CD4 and Functional Changes in African Green Monkey Memory CD4(+) T Cells. J. Immunol..

[28] Mudd J.C., Lai S., Shah S., Rahmberg A., Flynn J.K., Starke C.E., Perkins M.R., Ransier A., Darko S., Douek D.C. (2020). Epigenetic silencing of CD4 expression in nonpathogenic SIV infection in African green monkeys. JCI Insight.

[29] Mudd J.C., Perkins M.R., DiNapoli S.R., Hirsch V.M., Brenchley J.M. (2016). Interleukin-2 Therapy Induces CD4 Downregulation, Which Decreases Circulating CD4 T Cell Counts, in African Green Monkeys. J. Virol..

[30] Perkins M.R., Briant J.A., Calantone N., Whitted S., Vinton C.L., Klatt N.R., Ourmanov I., Ortiz A.M., Hirsch V.M., Brenchley J.M. (2014). Homeostatic cytokines induce CD4 downregulation in African green monkeys independently of antigen exposure to generate simian immunodeficiency virus-resistant CD8αα T cells. J. Virol..

[31] Vinton C.L., Ortiz A.M., Calantone N., Mudd J.C., Deleage C., Morcock D.R., Whitted S., Estes J.D., Hirsch V.M., Brenchley J.M. (2017). Cytotoxic T Cell Functions Accumulate When CD4 Is Downregulated by CD4(+) T Cells in African Green Monkeys. J. Immunol..

[32] Vinton C., Klatt N.R., Harris L.D., Briant J.A., Sanders-Beer B.E., Herbert R., Woodward R., Silvestri G., Pandrea I., Apetrei C. (2011). CD4-like immunological function by CD4- T cells in multiple natural hosts of simian immunodeficiency virus. J. Virol..

[33] Beaumier C.M., Harris L.D., Goldstein S., Klatt N.R., Whitted S., McGinty J., Apetrei C., Pandrea I., Hirsch V.M., Brenchley J.M. (2009). CD4 downregulation by memory CD4+ T cells in vivo renders African green monkeys resistant to progressive SIVagm infection. Nat. Med..

[34] Le Hingrat Q., Sette P., Xu C., Rahmberg A.R., Tarnus L., Annapureddy H., Kleinman A., Brocca-Cofano E., Sivanandham R., Sivanandham S. (2023). Prolonged experimental CD4(+) T-cell depletion does not cause disease progression in SIV-infected African green monkeys. Nat. Commun..

[35] Xu H., Wang X., Lackner A.A., Veazey R.S. (2013). CD8 down-regulation and functional impairment of SIV-specific cytotoxic T lymphocytes in lymphoid and mucosal tissues during SIV infection. J. Leukoc. Biol..

[36] DeMaster L.K., Liu X., VanBelzen D.J., Trinite B., Zheng L., Agosto L.M., Migueles S.A., Connors M., Sambucetti L., Levy D.N. (2015). A Subset of CD4/CD8 Double-Negative T Cells Expresses HIV Proteins in Patients on Antiretroviral Therapy. J. Virol..

[37] Cheney K.M., Kumar R., Purins A., Mundy L., Ferguson W., Shaw D., Burrell C.J., Li P. (2006). HIV type 1 persistence in CD4- /CD8- double negative T cells from patients on antiretroviral therapy. AIDS Res. Hum. Retroviruses.

[38] Marodon G., Warren D., Filomio M.C., Posnett D.N. (1999). Productive infection of double-negative T cells with HIV in vivo. Proc. Natl. Acad. Sci. USA.

[39] Korbi F., Zamali I., Rekik R., Ben Hmid A., Hidri M., Kammoun Rebai W., Jelili Z., Masmoudi S., Rahal S.K., Ben Ayed A. (2022). Double-negative T cells are increased in HIV-infected patients under antiretroviral therapy. Medicine.

[40] Briceno O., Peralta-Prado A., Garrido-Rodriguez D., Romero-Mora K., Chavez-Torres M., Pinto Cardoso S., Alvarado de la Barrera C., Reyes-Teran G., Avila-Rios S. (2023). Double-Negative T Cell Number and Phenotype Alterations Before and After Effective Antiretroviral Treatment in Persons Living with HIV. AIDS Res. Hum. Retroviruses.

[41] Liang Q., Jiao Y., Zhang T., Wang R., Li W., Zhang H., Huang X., Tang Z., Wu H. (2013). Double Negative (DN) [CD3(+)CD4(-)CD8(-)] T cells correlate with disease progression during HIV infection. Immunol. Investig..

[42] Lu X., Su B., Xia H., Zhang X., Liu Z., Ji Y., Yang Z., Dai L., Mayr L.M., Moog C. (2016). Low Double-Negative CD3(+)CD4(-)CD8(-) T Cells Are Associated with Incomplete Restoration of CD4(+) T Cells and Higher Immune Activation in HIV-1 Immunological Non-Responders. Front. Immunol..

[43] Meythaler M., Martinot A., Wang Z., Pryputniewicz S., Kasheta M., Ling B., Marx P.A., O'Neil S., Kaur A. (2009). Differential CD4+ T-lymphocyte apoptosis and bystander T-cell activation in rhesus macaques and sooty mangabeys during acute simian immunodeficiency virus infection. J. Virol..

[44] Favre D., Lederer S., Kanwar B., Ma Z.M., Proll S., Kasakow Z., Mold J., Swainson L., Barbour J.D., Baskin C.R. (2009). Critical loss of the balance between Th17 and T regulatory cell populations in pathogenic SIV infection. PLoS Pathog..

[45] Sundaravaradan V., Saleem R., Micci L., Gasper M.A., Ortiz A.M., Else J., Silvestri G., Paiardini M., Aitchison J.D., Sodora D.L. (2013). Multifunctional double-negative T cells in sooty mangabeys mediate T-helper functions irrespective of SIV infection. PLoS Pathog..

[46] Milush J.M., Mir K.D., Sundaravaradan V., Gordon S.N., Engram J., Cano C.A., Reeves J.D., Anton E., O'Neill E., Butler E. (2011). Lack of clinical AIDS in SIV-infected sooty mangabeys with significant CD4+ T cell loss is associated with double-negative T cells. J. Clin. Investig..

[47] Zhang L., Wei Y., Wang D., Du J., Wang X., Li B., Jiang M., Zhang M., Chen N., Deng M. (2022). Elevated Foxp3(+) double-negative T cells are associated with disease progression during HIV infection. Front. Immunol..

[48] Yero A., Farnos O., Clain J., Zghidi-Abouzid O., Rabezanahary H., Racine G., Estaquier J., Jenabian M.A. (2022). Impact of Early ARV Initiation on Relative Proportions of Effector and Regulatory CD8 T Cell in Mesenteric Lymph Nodes and Peripheral Blood During Acute SIV Infection of Rhesus Macaques. J. Virol..

[49] Yero A., Farnos O., Rabezanahary H., Racine G., Estaquier J., Jenabian M.A. (2019). Differential Dynamics of Regulatory T-Cell and Th17 Cell Balance in Mesenteric Lymph Nodes and Blood following Early Antiretroviral Initiation during Acute Simian Immunodeficiency Virus Infection. J. Virol..

[50] McLaughlin D., Faller E., Sugden S., MacPherson P. (2014). Expression of the IL-7 receptor alpha-chain is down regulated on the surface of CD4 T-cells by the HIV-1 Tat protein. PLoS ONE.

[51] Faller E.M., McVey M.J., MacPherson P.A. (2014). IL-7 receptor recovery on CD8 T-cells isolated from HIV+ patients is inhibited by the HIV Tat protein. PLoS ONE.

[52] Hasley R.B., Hong C., Li W., Friesen T., Nakamura Y., Kim G.Y., Park J.H., Hixon J.A., Durum S., Hu Z. (2013). HIV immune activation drives increased Eomes expression in memory CD8 T cells in association with transcriptional downregulation of CD127. AIDS.

[53] Jenabian M.A., Ancuta P., Gilmore N., Routy J.P. (2012). Regulatory T cells in HIV infection: Can immunotherapy regulate the regulator?. Clin. Dev. Immunol..

[54] Jenabian M.A., Seddiki N., Yatim A., Carriere M., Hulin A., Younas M., Ghadimi E., Kök A., Routy J.P., Tremblay A. (2013). Regulatory T cells negatively affect IL-2 production of effector T cells through CD39/adenosine pathway in HIV infection. PLoS Pathog..

[55] Yero A., Bouassa R.M., Ancuta P., Estaquier J., Jenabian M.A. (2023). Immuno-metabolic control of the balance between Th17-polarized and regulatory T-cells during HIV infection. Cytokine Growth Factor. Rev..

[56] Maartens G., Celum C., Lewin S.R. (2014). HIV infection: Epidemiology, pathogenesis, treatment, and prevention. Lancet.

[57] Rajasuriar R., Wright E., Lewin S.R. (2015). Impact of antiretroviral therapy (ART) timing on chronic immune activation/inflammation and end-organ damage. Curr. Opin. HIV AIDS.

[58] Crawley A.M., Angel J.B. (2012). The influence of HIV on CD127 expression and its potential implications for IL-7 therapy. Semin. Immunol..

[59] Yero A., Shi T., Farnos O., Routy J.P., Tremblay C., Durand M., Tsoukas C., Costiniuk C.T., Jenabian M.A. (2021). Dynamics and epigenetic signature of regulatory T-cells following antiretroviral therapy initiation in acute HIV infection. EBioMedicine.

[60] Masopust D., Vezys V., Marzo A.L., Lefrançois L. (2001). Preferential localization of effector memory cells in nonlymphoid tissue. Science.

[61] Sallusto F., Lenig D., Förster R., Lipp M., Lanzavecchia A. (1999). Two subsets of memory T lymphocytes with distinct homing potentials and effector functions. Nature.

[62] Francisco L.M., Salinas V.H., Brown K.E., Vanguri V.K., Freeman G.J., Kuchroo V.K., Sharpe A.H. (2009). PD-L1 regulates the development, maintenance, and function of induced regulatory T cells. J. Exp. Med..

[63] Chen X., Fosco D., Kline D.E., Meng L., Nishi S., Savage P.A., Kline J. (2014). PD-1 regulates extrathymic regulatory T-cell differentiation. Eur. J. Immunol..

[64] Asano T., Kishi Y., Meguri Y., Yoshioka T., Iwamoto M., Maeda Y., Yagita H., Tanimoto M., Koreth J., Ritz J. (2015). PD-1 signaling has a critical role in maintaining regulatory T cell homeostasis; implication for Treg depletion therapy by PD-1 blockade. Blood.

[65] Qureshi O.S., Zheng Y., Nakamura K., Attridge K., Manzotti C., Schmidt E.M., Baker J., Jeffery L.E., Kaur S., Briggs Z. (2011). Trans-endocytosis of CD80 and CD86: A molecular basis for the cell-extrinsic function of CTLA-4. Science.

[66] Sakaguchi S., Mikami N., Wing J.B., Tanaka A., Ichiyama K., Ohkura N. (2020). Regulatory T Cells and Human Disease. Annu. Rev. Immunol..

[67] Ma X., Cao L., Raneri M., Wang H., Cao Q., Zhao Y., Bediaga N.G., Naselli G., Harrison L.C., Hawthorne W.J. (2023). Human HLA-DR+CD27+ regulatory T cells show enhanced antigen-specific suppressive function. JCI Insight.

[68] Schulze Zur Wiesch J., Thomssen A., Hartjen P., Tóth I., Lehmann C., Meyer-Olson D., Colberg K., Frerk S., Babikir D., Schmiedel S. (2011). Comprehensive analysis of frequency and phenotype of T regulatory cells in HIV infection: CD39 expression of FoxP3+ T regulatory cells correlates with progressive disease. J. Virol..

[69] Nikolova M., Carriere M., Jenabian M.A., Limou S., Younas M., Kök A., Huë S., Seddiki N., Hulin A., Delaneau O. (2011). CD39/adenosine pathway is involved in AIDS progression. PLoS Pathog..

[70] (2009). Regulatory T Cell Expansion and Immune Activation during Untreated HIV Type 1 Infection Are Associated with Disease Progression. AIDS Res. Human. Retroviruses.

[71] Toth I., Le A.Q., Hartjen P., Thomssen A., Matzat V., Lehmann C., Scheurich C., Beisel C., Busch P., Degen O. (2013). Decreased frequency of CD73+CD8+ T cells of HIV-infected patients correlates with immune activation and T cell exhaustion. J. Leukoc. Biol..

[72] Wang X., Zhang L., Du J., Wei Y., Wang D., Song C., Chen D., Li B., Jiang M., Zhang M. (2022). Decreased CD73+ Double-Negative T Cells and Elevated Level of Soluble CD73 Correlated With and Predicted Poor Immune Reconstitution in HIV-Infected Patients After Antiretroviral Therapy. Front. Immunol..

