# Supplementary material for: Double-Negative T-Cells during Acute Human Immunodeficiency Virus and Simian Immunodeficiency Virus Infections and Following Early Antiretroviral Therapy Initiation
Source: Viruses. 2024 Oct 14;16(10):1609. doi: 10.3390/v16101609 (PMC11512404; doi:10.3390/v16101609)
Supplement: Supplementary file 1 [file viruses-16-01609-s001.zip › viruses-3216336-supplementary.pdf]

Table S1. Description of viral load in all human study participants.

ID.	Viral load (log10 copies/mL)		Viral load (copies/mL)	
	**Acute**	**ART-Treated**	**Acute**	**ART-Treated**
A1	4.00	2.40	10011	252
A2	4.20	1.70	15853	50
A3	6.94	1.70	8766394	50
A4	5.50	1.70	314458	50
A5	3.71	1.70	5103	50
A6	5.68	1.70	477916	50
A7	4.16	1.70	14562	50
A8	4.59	1.60	39334	40
A9	3.04	1.70	1091	50
A10	6.05	1.60	1124520	40
MEDIAN	4.40	1.70	27594	50
MEAN	4.79	1.75	1076924	68.20
STANDARD DEVIATION	1.21	0.23	2725313	64.71
IQR	3.93-5.77	1.68-1.70	8784-639567	47.5-50

IQR: interquartile range. *Nota bene*: Undetectable levels are defined at 50 or 40 copies/mL, depending on the assay's detection limit.

Table S2. Description of viral load in Rhesus macaques.

	Acute Untreated			Early ART-Treated		
	**ID.**	**Viral load (copies/mL)**	**Viral load (log10 copies/mL)**	**ID**	**Viral load (copies/mL)**	**Viral load (log10 copies/mL)**
	PB006	28000000	7.45	R110806	580	2.76
	PB041	1900000	6.28	11-1466R	40	1.60
	PB005	5800000	6.76	R110804	40	1.60
	PB051	47000000	7.67	R110562	40	1.60
	PB015	1100000	6.04	11-1430R	40	1.60
	PB033	6500000	6.81	R110360	40	1.60
	9051222	1600000	6.20	R110482	40	1.60
	PB044	710000	5.85	13-1660R	40	1.60
	PB023	5700000	6.76	12-1836R	40	1.60
	PB028	2300000	6.36			
	PB055	32000000	7.51			
	PB030	21000000	7.32			
	9082012	17000	4.23			
MEDIAN		5700000	6.76		40	1.6
MEAN		11817462	6.557		100	1.729
STANDARD DEVIATION		15176938	0.9173		180	0.3867
IQR		1350000-24500000	6.12-7.39		40-40	1.60-1.60

IQR: interquartile range. *Nota bene*: Undetectable levels are defined at 40 copies/mL.
